# Association between physical function and type 2 diabetes risk in older adults: a longitudinal analysis from the KoGES-Anseong cohort

**DOI:** 10.1007/s41999-026-01412-2

**Published:** 2026-07-06

**Authors:** Soomin Lee, DooYong Park, JinWon Rho, Jiwon Hong, Yeon Soo Kim

**Affiliations:** 1 Department of Physical Education, College of Education, Seoul National University, Seoul, Korea; 2 Department of Sport Science, Korea Institute of Sports Science, Seoul, Republic of Korea; 3 Institute of Sports Science, Seoul National University, 1 Gwanak-ro, Gwanak-gu, Seoul, 08826 Republic of Korea

**Keywords:** Physical fitness, Mobility limitation, Lower-extremity strength, Aged

## Abstract

**Purpose:**

Type 2 diabetes (T2D) is associated with functional decline in older adults, but whether baseline physical function predicts its incidence remains unclear. This study examined whether the Short Physical Performance Battery (SPPB) predicts incident T2D in older Koreans, and whether resting heart rate (RHR) modifies this association.

**Methods:**

We included 1,041 community-dwelling adults aged ≥ 65 years (mean age = 72.2 years; 58.3% women) without T2D at baseline from the KoGES-Anseong cohort (2013–2020). SPPB scores were categorized as high (≥ 10) or low (< 10), and RHR was dichotomized at the median. Incident T2D was ascertained through fasting glucose, physician diagnosis, or diabetes medication. Cox proportional hazards models (adjusted for demographic, metabolic, and lifestyle factors) estimated hazard ratios (HRs) and 95% confidence intervals (CIs) over 5294 person-years of follow-up.

**Results:**

High SPPB scores were associated with a lower risk of T2D (HR = 0.49, 95% CI 0.25–0.94). This association was observed only in participants with elevated RHR (HR = 0.37, 95% CI 0.17–0.81), but not in those with lower RHR. Among individual SPPB domains, only lower-extremity strength showed a significant association with reduced T2D risk.

**Conclusion:**

In older adults, superior physical function substantially reduces the risk of T2D, with lower-extremity strength as the primary contributor. Importantly, this benefit remains robust among individuals with elevated RHR, underscoring functional performance as a practical target for preventive strategies.

**Supplementary Information:**

The online version contains supplementary material available at https://doi.org/10.1007/s41999-026-01412-2.

## Key summary points

**Aim:**

To examine whether physical function, assessed by the Short Physical Performance Battery (SPPB), predicts incident type 2 diabetes (T2D) in older adults, and whether resting heart rate (RHR) modifies this association.

**Findings:**

Higher SPPB scores were associated with a significantly lower risk of T2D, with the protective effect observed only among participants with elevated RHR. Among the SPPB components, lower-extremity strength was the most relevant predictor.

**Message:**

Routine functional assessments may provide a practical strategy for early identification and prevention of T2D in older adults, particularly those with elevated RHR who face increased metabolic vulnerability.

**Supplementary Information:**

The online version contains supplementary material available at https://doi.org/10.1007/s41999-026-01412-2.

## Introduction

Type 2 diabetes (T2D) is a chronic metabolic disorder characterized by impaired insulin secretion and/or action, and constitutes a major global public health challenge, particularly within the aging population [1, 2]. The aging process combines risk factors such as metabolic deterioration, reduced physical activity, and multimorbidity, which substantially increase susceptibility to T2D and identify older adults as a critical target for prevention [3, 4]. This burden is especially pronounced in nations with rapidly aging populations, such as South Korea. Nationally representative health statistics indicate that the prevalence of T2D is 15.5% among adults aged ≥ 30 years, and that approximately one in three adults aged ≥ 65 years is affected [5, 6].

This high prevalence is clinically important because T2D accelerates functional decline, thereby increasing the risk of loss of independence in older adults [7–9]. While glycemic control and complication management remain central to T2D care, there is increasing recognition that, particularly for older adults, effective management should also encompass the preservation of physical function [10–12]. This shift reflects an evolving view of successful aging, defined not only by the absence of complications but also by the capacity to maintain independence in daily activities rather than focusing solely on the prevention of clinical complications [13]. In line with this shift, identifying older adults at risk of functional decline has gained attention as a clinical priority, underscoring the need for a valid and feasible assessment tool.

The Short Physical Performance Battery (SPPB), a validated measure of balance, gait speed, and lower-extremity strength, is widely used to assess functional status and predict adverse outcomes such as disability, hospitalization, and mortality [14, 15]. However, its role as a prospective predictor of incident T2D remains underexplored. Emerging evidence suggests that resting heart rate (RHR), a marker of autonomic imbalance and cardiometabolic stress, is associated with poorer physical function and accelerated functional decline. This raises the possibility that physiological dysregulation may heighten susceptibility to metabolic deterioration among individuals with lower functional performance. Although elevated RHR has been linked to increased T2D risk [16], it is unclear whether autonomic function modifies the association between physical performance and T2D development [17, 18].

Therefore, this study aimed to investigate the prospective association between physical function, as measured by the SPPB, and the risk of incident T2D in a community-dwelling older Korean population, and to examine whether this association is modified by RHR. Accordingly, we hypothesized that higher physical function would be associated with a lower risk of developing T2D, with a more pronounced protective effect among individuals with elevated RHR.

## Method

### Study population

We conducted a prospective cohort analysis using data from the Anseong cohort, a regional subset of the Korean Genome and Epidemiology Study (KoGES), a nationwide, population-based longitudinal study designed to investigate chronic disease risk factors in Korean adults [19]. Among 5645 individuals who participated in the health examination, 4192 participants had at least two repeated assessments between 2013 and 2020 and were therefore eligible for follow-up, and had complete measurements for SPPB, RHR and all covariates. Of the 4192 individuals, we excluded 2933 participants based on baseline criteria, including those who were younger than 65 years (n = 1269), had prevalent T2D at baseline (n = 1617), or reported physician-diagnosed cardiovascular disease (n = 23) or cancer (n = 24). Incident T2D cases were identified only among participants who were non-diabetic at baseline and were defined during follow-up using fasting glucose levels, physician diagnosis, or use of diabetes medication, ensuring that all cases represented new-onset T2D. An additional 218 participants were excluded due to missing data on primary outcomes or covariates (age, sex, physical activity, body mass index (BMI), skeletal muscle mass, sleep duration, estimated glomerular filtration rate (eGFR), high-sensitivity C-reactive protein (hs-CRP), and homeostasis model assessment of insulin resistance (HOMA-IR), triglycerides, total cholesterol, alcohol consumption, smoking status, income level, and RHR). The final analytic cohort consisted of 1041 participants.

Ethical approval for this study was obtained from an institutional review board (IRB) at Seoul National University in South Korea (IRB No. E2112/001-009). All participants received a thorough explanation of the study objectives and procedures and then provided written informed consent.

### Assessment of variables

#### T2D

The diagnosis of T2D was based on a previous study on T2D classification and management published in 2023, as well as a study examining RHR, relative grip strength, and T2D prevalence [20, 21]. Participants were classified as having T2D if they had a fasting blood glucose level of ≥ 126 mg/dL, or if they responded “yes” to either of the following questions: “Have you ever been diagnosed with T2D?” or “Are you currently taking medication for T2D?”. T2D status was assessed repeatedly at each follow-up examination, and incident T2D was defined as a new diagnosis occurring after baseline among participants free of T2D at baseline.

#### SPPB score

SPPB is a reliable tool for evaluating physical function and frailty including balance, lower-extremity strength, and walking speed among older adults. SPPB consists of three subsets: (1) balance: assessment of maintaining balance in three different foot positions, (2) lower-extremity strength: measurement of time required to complete sit-to-stand position five times consecutively and (3) walking speed: measurement of time taken to walk 4 m.

Each subtest is scored from 0 to 4, with the total score ranging from 0 to 12. A higher SPPB score reflects better physical functioning, whereas a lower score may indicate physical impairment or increased frailty risk. In addition to assessing physical function, the SPPB has been shown to predict a range of adverse health outcomes, including falls, mobility decline, functional disability, loss of independence in activities of daily living and mortality. In this study, participants were categorized into two groups based on total SPPB score: SPPB High (score ≥ 10) and SPPB Low (score < 10) [22].

#### Laboratory measurements

All participants fasted for at least 8 h prior to blood sample collection. On the examination day, blood samples were collected, centrifuged on-site, and sent to the Seoul Clinical Laboratory (Seoul, Korea) for analysis using the ADVIA 1800 auto analyzer (Siemens, USA). The results related to T2D including blood glucose, hs-CRP, serum creatinine, insulin, triglycerides, and total cholesterol, were included in this study. Serum samples were stored by the Genomic Epidemiology Department at the Korea Disease Control and Prevention Agency. The eGFR was calculated using the MDRD study equation (in mL/min per 1.73 m^2^ = 175 × SCr-1.154 × age-0.203 × [0.742 if female]), based on serum creatinine values, and treated as a continuous variable [20].

#### Questionnaire and other variables

This study included various covariates including physical measurements, lifestyle factors, and biochemical markers. Height (cm) and weight (kg) were each measured once. BMI (kg/m^2^) was calculated by dividing weight (kg) by the square of height (m^2^). Lean body mass was measured using a bioelectrical impedance analysis (InBody 3.0, Biospace, Korea) and was used to adjust for muscle strength and metabolism related variables. RHR was measured using an automatic blood pressure monitor and was defined as the beats per minute recorded during blood pressure measurement. RHR was divided into two groups based on the median value.

Lifestyle and socioeconomic variables were collected through face-to-face interviews conducted by trained surveyors. To ensure the accuracy and consistency of responses, surveys were reviewed and supplemented on the day of the interview. Alcohol consumption was categorized as never, former, or current based on responses to the question, “Have you never been able to drink alcohol, or have you chosen not to drink from the beginning?” and follow-up questions regarding current drinking status. Smoking status was classified as never smoker, former smoker, or current smoker based on participants' responses to the question, “Have you ever smoked more than five packs (100 cigarettes) in your lifetime?” and a follow-up question “Do you currently smoke?”. Engagement in regular physical activity was categorized as “regular exercise participation” and ‘non-participation’ based on responses to the question “Do you regularly engage in exercise that makes you sweat?”. Monthly household income was categorized into five levels: “ < 1,000,000 KRW”, “1,000,000–1,990,000 KRW”, “2,000,000–2,990,000 KRW”, “3,000,000–3,990,000 KRW”, “ ≥ 4,000,000 KRW”.

#### Statistical analysis

All analyses were performed using STATA/IC version 14.1 (StataCorp., College Station, TX, USA). Descriptive statistics were used for continuous variables, presented as mean ± SD to summarize demographic characteristics. Categorical variables were analyzed using chi-square tests and presented as frequencies and percentages (%). Additionally, one-way ANOVA was conducted to examine differences in variables according to SPPB levels and RHR categories, followed by Bonferroni post-hoc tests (p < 0.05).

Incidence density of T2D was calculated using person-years accumulated over the entire follow-up period. To assess the association between baseline SPPB levels (Low, High) and the incidence of T2D, multivariable extended Cox proportional hazards models were performed to estimate the independent hazard ratios (HRs) and 95% confidence intervals (CIs). The same model was applied to assess the association between RHR levels (Low, High) and T2D risk within each SPPB level. In addition, the association between each individual component of the SPPB and T2D risk was evaluated using the same analytical approach.

In the multivariable extended Cox proportional hazards Model 1 was adjusted for age and sex. Model 2 was additionally adjusted for sleep duration, skeletal muscle mass, triglycerides, total cholesterol, eGFR, hs-CRP, alcohol intake, smoking status, income level, HOMA-IR, and participation in regular exercise. Model 3 was further adjusted for RHR in addition to all variables included in Models 1 and 2. All statistical significance was set at p < 0.05. All covariates, including SPPB and RHR, were measured at baseline and treated as time-independent (fixed) variables in all analyses.

## Results

### Baseline characteristics of the study population

Baseline characteristics of the study participants, stratified by SPPB level, are presented in Table 1. Of the 1,041 participants, 113 (10.9%) were classified into the low SPPB group and 928 (89.1%) into the high SPPB group. Compared to the low SPPB group, participants in the high SPPB group were significantly younger (72.0 ± 4.5 vs. 75.6 ± 3.4 years, p < 0.001) and had a higher proportion of males (43.0% vs. 20.4%, p < 0.001). The high SPPB group also demonstrated greater lean body mass (38.3 ± 7.1 vs. 34.5 ± 6.1 kg, p < 0.001) and higher eGFR (91.6 ± 21.1 vs. 85.8 ± 21.0 mL/min per 1.73m^2^, p = 0.002). In terms of lifestyle factors, participants with high SPPB were more likely to engage in regular exercise (24.6% vs. 10.6%, p = 0.001), but also had a higher rate of current smoking (9.8% vs. 4.4%, p = 0.002). There were no statistically significant differences between the two groups in BMI, RHR, sleep duration, hs-CRP, HOMA-IR, lipid profiles (triglycerides, total cholesterol), alcohol consumption, or low-income status. Baseline characteristics were also analyzed according to RHR level **(**Table 2**)**. Of the participants, 343 were in the low RHR group and 698 were in the high RHR group. Overall, the high RHR group exhibited a less favorable cardiometabolic profile. Specifically, they had significantly higher levels of hs-CRP (1.9 vs. 1.3 mg/L, p = 0.021), triglycerides (129.3 vs. 116.7 mg/dL, p = 0.007), total cholesterol (183.4 vs. 179.2 mg/dL, p = 0.042), and HOMA-IR (2.0 vs. 1.8, p = 0.004). In terms of body composition and demographics, the high RHR group had lower lean body mass (37.3 vs. 39.0 kg, p < 0.001) and a lower proportion of males (36.8% vs. 48.1%, p < 0.001). The prevalence of current smoking was also lower in the high RHR group (8.7% vs. 10.2%, p < 0.001). There were no significant between-group differences in age, BMI, sleep duration, eGFR, current alcohol consumption, regular exercise, low-income status, or the proportion of participants with low SPPB scores. Details of the participant selection process are provided in Supplementary Figure S1**.**

**Table 1:** Baseline characteristics of study participants by SPPB level

Characteristics of risk factor	Total (n = 1031)	Low SPPB score (n = 113)	High SPPB score (n = 928)	p-value
Fasting glucose (mg/dL)	92.0 ± 9.5	90.0 ± 9.4	92.2 ± 9.5	0.018
Age (years)	75.6 ± 3.9	72.0 ± 4.5	< 0.001	75.6 ± 3.9
Sex (Male %)	20.4	43.0	< 0.001	20.4
BMI (kg/m^2^)	24.1 ± 4.2	23.9 ± 3.2	0.428	24.1 ± 4.2
Lean body mass (kg)	34.5 ± 6.1	38.3 ± 7.1	< 0.001	34.5 ± 6.1
RHR(beat/min)	62.6 ± 8.4	61.5 ± 7.6	0.138	62.6 ± 8.4
Sleep duration (hr/day)	6.6 ± 1.4	6.6 ± 1.3	0.885	6.6 ± 1.4
eGFR (mL/min per 1.73m^2^)	85.2 ± 21.1	91.6 ± 21.1	0.002	85.2 ± 21.1
hs-CRP (mg/dL)	1.5 ± 2.1	1.7 ± 4.3	0.540	1.5 ± 2.1
Triglyceride (mg/dL)	121.9 ± 49.0	125.5 ± 73.0	0.612	121.9 ± 49.0
Total Cholesterol (mg/dL)	181.5 ± 34.9	182.1 ± 31.4	0.851	181.5 ± 34.9
HOMA-IR	2.0 ± 1.1	2.0 ± 1.1	0.833	2.0 ± 1.1
Current alcohol consumption (%)	24.8	33.4	0.167	24.8
Current smoking (%)	4.4	9.8	0.002	4.4
Regular exercise participation (%)	10.6	24.6	0.001	10.6
Low income status (< 1 million won %)	79.6	71.7	0.362	79.6
SPPB score				
Balance	3.2 ± 0.7	3.9 ± 0.3	< 0.001	3.2 ± 0.7
Sit to stand	2.7 ± 1.0	3.9 ± 0.3	< 0.001	2.7 ± 1.0
Walking speed	2.2 ± 0.8	3.5 ± 0.6	< 0.001	2.2 ± 0.8

*BMI* body mass index, *eGFR* estimated glomerular filtration rate, *HOMA-IR* homeostatic model assessment for insulin resistance, *hs-CRP* human serum-C-reactive protein, *RHR* rest heart rate, *SPPB* short physical performance battery

**Table 2:** Baseline characteristics of study participants by RHR level

Characteristics of risk factor	Total (n = 1031)	Low RHR (n = 343)	High RHR (n = 698)	p-value
Range of RHR (min ~ max)	44 ~ 98	44 ~ 58	60 ~ 98	–
Fasting Glucose (mg/dL)	92.0 ± 9.5	91.5 ± 9.2	92.2 ± 9.6	0.281
Low SPPB Score (%)	10.8	11.1	10.7	0.871
Age (years)	72.2 ± 4.5	72.5 ± 4.6	72.4 ± 4.5	0.850
Sex (Male %)	41.7	48.1	36.8	< 0.001
BMI (kg/m^2^)	23.9 ± 3.3	23.9 ± 3.3	23.9 ± 3.4	0.968
Lean body mass (kg)	38.1 ± 7.1	39.1 ± 7.0	37.3 ± 7.1	< 0.001
Sleep duration (hr/day)	6.6 ± 1.3	6.6 ± 1.4	6.5 ± 1.3	0.221
eGFR (mL/min per 1.73m^2^)	90.8 ± 21.5	90.2 ± 20.4	91.2 ± 21.6	0.470
hs-CRP (mg/dL)	1.8 ± 4.3	1.3 ± 1.7	1.9 ± 4.8	0.021
Triglyceride (mg/dL)	125.6 ± 72.2	116.7 ± 59.0	129.3 ± 75.6	0.007
Total cholesterol(mg/dL)	181.6 ± 31.9	179.2 ± 31.4	183.4 ± 31.9	0.042
HOMA-IR	2.0 ± 1.1	1.8 ± 0.8	2.0 ± 1.2	0.004
Current alcohol consumption (%)	33.0	33.8	31.8	0.115
Current smoking (%)	8.8	10.2	8.7	< 0.001
Regular exercise participation (%)	13.9	25.4	21.9	0.215
Low income status (< 1 million won %)	72.3	73.3	72.1	0.198

*BMI* body mass index, *eGFR* estimated glomerular filtration rate, *HOMA-IR* homeostatic model assessment for insulin resistance, *hs-CRP* human serum-C-reactive protein, *RHR* rest heart rate, *SPPB* short physical performance battery

### Association between SPPB and incident T2D

Table 3 shows the association between SPPB score levels and the incidence of T2D. During 5294.9 person-years of follow-up, the incidence rate was 23.2 per 1000 person-years in the lower SPPB group and 16.5 per 1000 person-years in the higher SPPB group. In Model 1, adjusted for age and sex, participants in the higher SPPB group had a significantly lower risk of developing T2D compared with those in the lower group (HR = 0.48, 95% CI 0.25–0.93). This association remained consistent after further adjustment for lifestyle and metabolic factors in Model 2 (HR = 0.49, 95% CI 0.25–0.94) and Model 3, which additionally adjusted for RHR (HR = 0.49, 95% CI 0.25–0.94).

**Table 3:** Incidence density and hazard ratio of Type 2 diabetes according to SPPB Score by RHR level

Characteristics of risk factors		T2B	Person-year	Incidence density† (95% CI)	Model 1 (95% CI)	Model 2 HR (95% CI)	Model 3 HR (95% CI)
Total (n = 1041)							
SPPB	Low	12	517.0	23.2 (13.2, 40.9)	1.00 (Reference)	1.00 (Reference)	1.00 (Reference)
	High	79	4777.9	16.(13.3, 20.6)	0.48 (0.25, 0.93)	0.49 (0.25, 0.94)	0.49 (0.25, 0.94)
Total (Total)		91	5294.93	17.2 (14.0, 21.1)	**–**	**–**	**–**
Low RHR (n = 343)							
SPPB	Low	3	182.4	16.4 (5.3, 51.0)	1.00 (Reference)	1.00 (Reference)	**–**
	High	26	1555.1	16.7 (11.4, 24.6)	0.63 (0.17, 2.29)	1.15 (0.28, 4.73)	–
Total (Low RHR)		29	1737.6	16.7 (11.6, 24.0)	–	–	–
High RHR (n = 698)							
SPPB	Low	9	334.6	26.9 (14.0, 51.7)	1.00 (Reference)	1.00 (Reference)	–
	High	53	3222.8	16.4 (12.6, 21.5)	0.44* (0.20, 0.93)	0.37* (0.17, 0.81)	–
Total (High RHR)		62	3557.4	17.4 (13.6, 22.4)	–	–	–

^†^Incidence density = case/person-year × 1000

Multivariable Model 1 was adjusted for age and sex. Model 2 was additionally adjusted for sleep duration, lean body mass, triglycerides, total cholesterol, eGFR, hs-CRP, alcohol intake, smoking status, income status, HOMA-IR, and exercise participation. Model 3 was further adjusted for RHR in addition to all variables in Models 1 and 2

*BMI* body mass index, *T2B* type 2 diabetes mellitus,* eGFR* estimated glomerular filtration rate, *HOMA-IR* homeostatic model assessment for insulin resistance, *HR* hazard ratio,* hs-CRP* human serum-C-reactive protein, *RHR* rest heart rate, *SPPB* short physical performance battery

*p < 0.05, **p < 0.01, ***p < 0.001

### Subgroup analysis stratified by RHR

Among participants with lower RHR (n = 343; 1737.55 person-years), the incidence rates were 16.44 and 16.71 per 1000 person-years in the lower and higher SPPB groups, respectively, and no significant association was observed (Model 1: HR = 0.63, 95% CI 0.17–2.29; Model 2: HR = 1.15, 95% CI 0.28–4.73). In contrast, among participants with higher RHR (n = 698; 3557.38 person-years), the incidence rate was 26.90 per 1000 person-years in the lower SPPB group and 16.44 per 1000 person-years in the higher SPPB group. In this subgroup, the higher SPPB group showed a significantly lower risk of developing T2D (Model 1: HR = 0.44, 95% CI 0.20–0.93; Model 2: HR = 0.37, 95% CI 0.17–0.81).

### Association of individual SPPB components with incident T2D

**Fig. 1:**
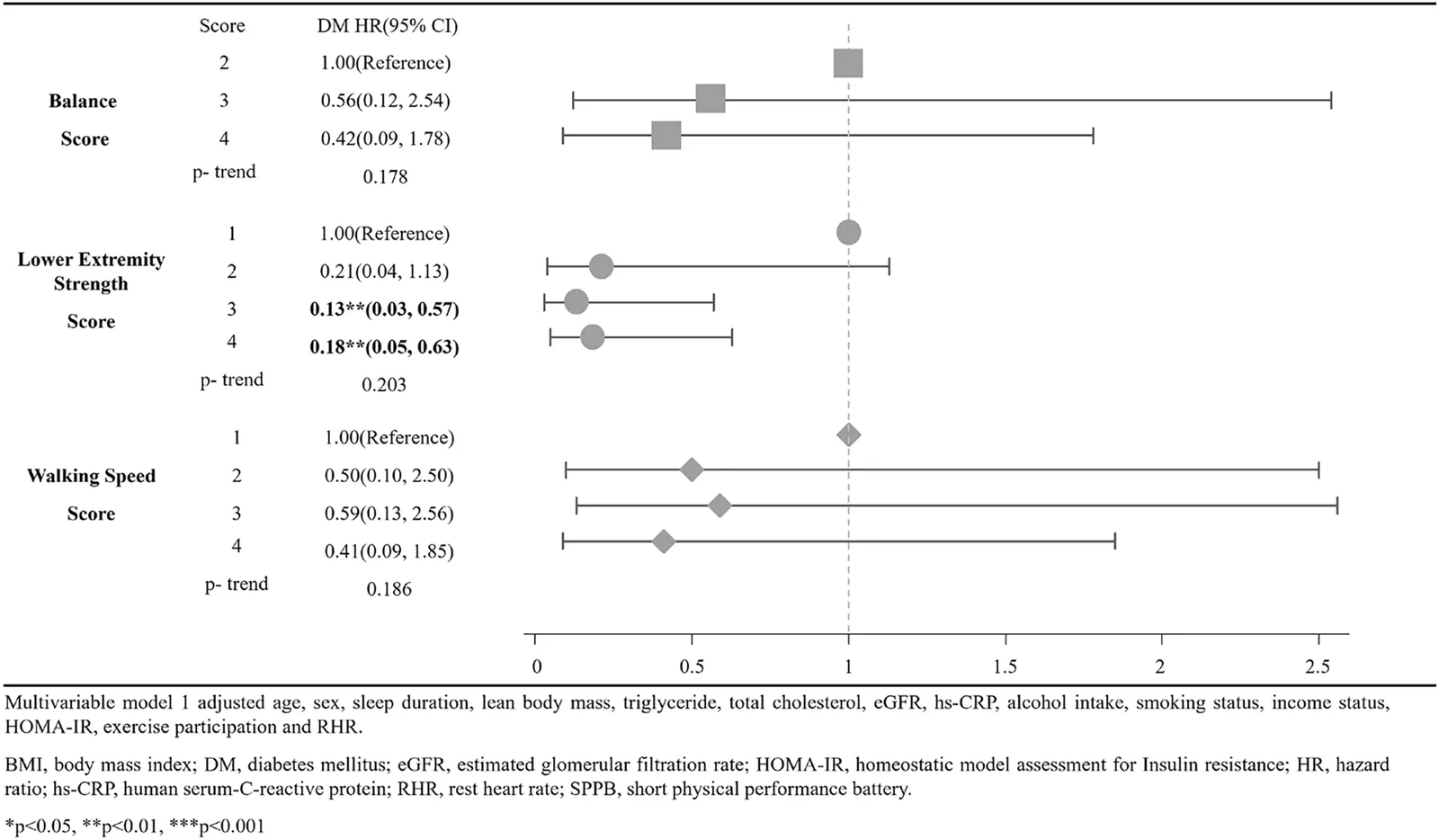
Hazard ratio of DM according to association between physical function by type of SPPB factors

Figure 1 illustrates the association between each SPPB component and the risk of developing T2D. Using a lower-extremity strength score of 1 as the reference, participants with a score of 3 had an 87% lower risk of developing T2D (HR = 0.13, 95% CI 0.03–0.57), and those with a score of 4 had an 82% lower risk (HR = 0.18, 95% CI 0.05–0.36). In contrast, neither balance performance nor gait speed showed significant associations with T2D risk.

## Discussion

In this prospective cohort of older adults, higher physical function assessed by the SPPB was significantly associated with a lower risk of incident T2D. This association was evident among individuals with elevated RHR, suggesting that higher physical function may attenuate T2D risk even in the presence of autonomic dysregulation. Among the individual SPPB components, lower-extremity strength measured by the chair-stand test was significantly associated with reduced diabetes risk. Our findings suggest that even among older adults with signs of autonomic dysregulation, higher physical function as assessed by the SPPB is associated with a lower risk of incident T2D. Within this context, the stronger association observed for the chair-stand component may reflect the relevance of lower-extremity function as one dimension of overall physical function.

Prior studies have consistently documented that T2D accelerates functional decline and reduces quality of life in older adults [23, 24]. Much of this work, however, has conceptualized physical function as a consequence of diabetes, showing that patients with T2D experience greater declines in mobility, muscle strength, and independence [8, 11]. By contrast, relatively few investigations have considered whether impairments in physical function *precede* the onset of T2D, thereby acting as a potential risk factor. This gap limits our understanding of whether objective tests like the SPPB can serve as early indicators of diabetes risk in aging populations.

As the largest site of glucose disposal, skeletal muscle is central to insulin sensitivity; thus, declines in muscle strength and function may impair glycemic regulation independent of muscle mass [25, 26]. Lower-extremity strength is especially relevant, representing the muscular and neuromuscular capacity underlying walking and daily activities that drive considerable metabolic demand [27]. Myokines released during muscle contraction, including interleukin-6 and irisin, have been implicated in anti-inflammatory pathways, lipid metabolism, and insulin signaling, supporting the biological plausibility that superior functional capacity may mitigate T2D risk [28]. Consistent with these mechanisms, longitudinal evidence indicates that reduced appendicular muscle mass more than doubles the risk of T2D, with each 1 SD decrement linked to an approximately 35% increase in risk [29]. This literature has emphasized structural parameters such as muscle quantity or behavioral indicators such as muscle mass, with relatively few studies evaluating whether performance-based measures can independently predict T2D in older populations [30–32]. This gap highlights the need to consider functional performance as an additional dimension of metabolic health in aging. In this context, our findings suggest that functional capacity may contribute to metabolic resilience, potentially mitigating the dual burden of muscle decline and autonomic dysregulation.

While these mechanisms explain the overall association between physical function and T2D risk, they do not fully account for why this association was particularly evident among individuals with elevated RHR. Elevated RHR is a well-recognized marker of autonomic imbalance and has been independently associated with an increased risk of T2D [16, 18]. It is often interpreted as a surrogate of sympathetic overactivity, which can promote insulin resistance, impair glucose uptake, and contribute to hyperglycemia [33]. Most previous studies have examined RHR only as an independent predictor of T2D, typically adjusting for adiposity, fitness, or physical activity. Few, however, have investigated whether RHR modifies the association between physical function and diabetes risk [17, 18, 34, 35] Building on this gap, we evaluated effect modification by RHR a priori. In stratified analyses, higher SPPB scores were associated with lower T2D incidence among participants with elevated RHR. One plausible explanation is that higher physical function, as captured by the SPPB, reflects preserved peripheral functional capacity and neuromuscular integration [36], which may partially buffer the metabolic stress associated with autonomic dysregulation [37]. Although elevated RHR is indicative of sympathetic overactivity and increased cardiometabolic risk, individuals with better functional performance may maintain greater insulin-independent glucose uptake, more efficient neuromuscular coordination, and higher daily energy expenditure through functional movement [38]. Collectively, these factors may contribute to metabolic resilience, thereby attenuating the risk of incident T2D even in the presence of elevated RHR.

While the present study adds to the growing evidence on the role of physical function in T2D risk among older adults, several limitations should be considered. First, although multiple metabolic and behavioral covariates were adjusted for, we were unable to include HbA1c or postprandial glucose levels, which may have affected the accuracy of diabetes ascertainment. Future studies should incorporate additional clinical markers to improve diagnostic validity. Second, dietary factors that could influence diabetes risk were not assessed. Further research is needed to explore how nutritional intake interacts with physical function in predicting T2D onset. Third, the study sample consisted of older adults from a single geographic region in South Korea, which may limit the generalizability of the findings to broader populations. Nationally representative cohorts would strengthen external validity. Lastly, physical function was measured at a single time point. Longitudinal assessments are warranted to clarify how changes in functional performance over time relate to metabolic health.

These findings underscore the potential role of functional capacity as a modifiable determinant of T2D risk. Importantly, the protective association was most pronounced among individuals with elevated RHR, suggesting that maintaining functional capacity may help offset metabolic vulnerability linked to autonomic imbalance. Within this context, lower-extremity strength emerged as the key component, underscoring its particular relevance for T2D in older adults. Given its feasibility and prognostic value, the SPPB may serve as a practical screening tool for early identification of older adults at high risk. From a public health perspective, integrating functional assessments into community-level prevention programs could facilitate tailored interventions that promote muscle strength, support autonomic regulation, and ultimately reduce the burden of T2D in aging populations.

## Supplementary Information

Below is the link to the electronic supplementary material.

Supplementary file1 (DOCX 61 KB)

## Data Availability

The data that support the findings of this study are available from a third-party national health repository. Restrictions apply to the availability of these data, which were used under license for the current study, and so are not publicly available. Information on data access can be provided by the authors upon reasonable request, subject to a data use agreement with the repository.

## Declarations

### Patient and public involvement

No patients or members of the public were involved in the design, conduct, reporting, or dissemination of this research.

## References

[1] Bellary S, Kyrou I, Brown JE, Bailey CJ (2021) Type 2 diabetes mellitus in older adults: clinical considerations and management. Nat Rev Endocrinol 17(9):534–548. 10.1038/s41574-021-00512-2 34172940

[2] ElSayed NA, Aleppo G, Aroda VR, Bannuru RR, Brown FM, Bruemmer D, Collins BS, Gaglia JL, Hilliard ME, Isaacs D (2023) Classification and diagnosis of diabetes: Standards of care in diabetes—2023. Diabetes Care 46(Suppl 1):S19-40. 10.2337/dc23-S002 36507649 PMC9810477

[3] Nanayakkara N, Curtis AJ, Heritier S, Gadowski AM, Pavkov ME, Kenealy T, Owens DR, Thomas RL, Song S, Wong J (2021) Impact of age at type 2 diabetes mellitus diagnosis on mortality and vascular complications: systematic review and meta-analyses. Diabetologia 64(2):275–287. 10.1007/s00125-020-05319-w 33313987 PMC7801294

[4] Hanlon P, Fauré I, Corcoran N, Butterly E, Lewsey J, McAllister D, Mair FS (2020) Frailty measurement, prevalence, incidence, and clinical implications in people with diabetes: a systematic review and study-level meta-analysis. Lancet Healthy Longev 1(3):e106–e116. 10.1016/S2666-7568(20)30014-3 33313578 PMC7721684

[5] Park SE, Ko SH, Kim JY, Kim K, Moon JH, Kim NH, Han KD, Choi SH, Cha BS (2025) Diabetes fact sheets in Korea 2024. Diabetes Metab J 49(1):2–4. 10.4093/dmj.2024.081 PMC11788544 39828976

[6] Zhou B, Rayner AW, Gregg EW, Sheffer KE, Carrillo-Larco RM, Bennett JE, Shaw JE, Paciorek CJ, Singleton RK, Pires AB (2024) Worldwide trends in diabetes prevalence and treatment from 1990 to 2022: a pooled analysis of 1108 population-representative studies with 141 million participants. Lancet 404(10436):2077–2093. 10.1016/S0140-6736(24)02317-1 39549716 PMC7616842

[7] Jing X, Chen J, Dong Y, Han D, Zhao H, Wang X, Gao F, Li C, Cui Z, Liu Y (2018) Related factors of quality of life of type 2 diabetes patients: a systematic review and meta-analysis. Health Qual Life Outcomes 16:189. 10.1016/S0140-6736(24)02317-1 30231882 PMC6147036

[8] Wong E, Backholer K, Gearon E, Harding J, Freak-Poli R, Stevenson C, Peeters A (2013) Diabetes and risk of physical disability in adults: a systematic review and meta-analysis. Lancet Diabetes Endocrinol 1(2):106–114. 10.1016/S2213-8587(13)70046-9 24622316

[9] Shang Y, Fratiglioni L, Vetrano DL, Dove A, Welmer AK, Xu W (2021) Not only diabetes but also prediabetes leads to functional decline and disability in older adults. Diabetes Care 44(3):690–698. 10.2337/dc20-2232 33446522 PMC7896268

[10] Harrington D, Henson J (2021) Physical activity and exercise in the management of type 2 diabetes: where to start? Pract Diabetes 38(2):35–40b. 10.1002/pdi.2361

[11] Ahmad E, Sargeant JA, Yates T, Webb DR, Davies MJ (2022) Type 2 diabetes and impaired physical function: a growing problem. Diabetology 3:30–45. 10.3390/diabetology3010003

[12] Cheval B, Maltagliati S, Sieber S, Beran D, Chalabaev A, Sander D, Cullati S, Boisgontier MP (2021) Why are individuals with diabetes less active? The mediating role of physical, emotional, and cognitive factors. Ann Behav Med 55(10):904–917. 10.1093/abm/kaaa120 33491067 PMC8382143

[13] Zhang J, Tam WS, Hounsri K, Kusuyama J, Wu VX (2024) Effectiveness of combined aerobic and resistance exercise on cognition, metabolic health, physical function, and health-related quality of life in middle-aged and older adults with type 2 diabetes mellitus: a systematic review and meta-analysis. Arch Phys Med Rehabil 105(8):1585–1599. 10.1016/j.apmr.2023.10.005 37875170

[14] Houston DK, Neiberg RH, Miller ME, Hill JO, Jakicic JM, Johnson KC, Gregg EW, Hubbard VS, Pi-Sunyer X, Rejeski WJ (2018) Physical function following a long-term lifestyle intervention among middle-aged and older adults with type 2 diabetes: the Look AHEAD study. J Gerontol A Biol Sci Med Sci 73(11):1552–1559. 10.1093/gerona/glx204 29053861 PMC6175031

[15] Jang HC (2019) Diabetes and muscle dysfunction in older adults. Ann Geriatr Med Res 23(4):160–164. 10.4235/agmr.19.0038 32743306 PMC7370755

[16] Kim G, Lee YH, Jeon J, Bang H, Lee BW, Kang E, Lee IK, Cha BS, Kim C (2017) Increase in resting heart rate over 2 years predicts incidence of diabetes: a 10-year prospective study. Diabetes Metab 43(1):25–32. 10.1016/j.diabet.2016.09.002 27745827

[17] Carnethon MR, Yan L, Greenland P, Garside DB, Dyer AR, Metzger B, Daviglus ML (2008) Resting heart rate in middle age and diabetes development in older age. Diabetes Care 31(2):335–339. 10.2337/dc07-0874 17959868

[18] Lee DH, de Rezen LFM, Hu FB, Jeon JY, Giovannucci EL (2019) Resting heart rate and risk of type 2 diabetes: a prospective cohort study and meta-analysis. Diabetes Metab Res Rev 35(2):e3095. 10.1002/dmrr.3095 30378246 PMC6398339

[19] Kim Y, Han BG, KoGES Group (2017) Cohort profile: the Korean genome and epidemiology study (KoGES) consortium. Int J Epidemiol 46(2):e20. 10.1093/ije/dyv316 27085081 PMC5837648

[20] Park DH, Han SH, Cho W, Jeon JY (2020) Higher resting heart rate and lower relative grip strength are associated with increased risk of diabetes in the Korean elderly population: Korean National Health and Nutrition Examination Survey 2015–2018. J Exerc Sci Acad 29(4):416–426. 10.15857/KSEP.2020.29.4.416

[21] Yang X, Sun J, Zhang W (2024) Global trends in burden of type 2 diabetes attributable to physical inactivity across 204 countries and territories, 1990-2019. Front Endocrinol 15:1343002. 10.3389/fendo.2024.1343002 PMC10925666 38469145

[22] Kwon S, Perera S, Pahor M, Katula J, King A, Groessl E, Studenski S (2009) What is a meaningful change in physical performance? Findings from a clinical trial in older adults (the LIFE-P study). J Nutr Health Aging 13(6):538–544. 10.1007/s12603-009-0104-z 19536422 PMC3100159

[23] Eusepi D, Pellicciari L, Ugolini A, Graziani L, Coppari A, Carlizza A, Caselli S, La Porta F, Paci M, Di Bari M (2025) Reliability of the short physical performance battery (SPPB): a systematic review with meta-analysis. Eur Geriatr Med 16:1–16. 10.1007/s41999-025-01277-x 40679769

[24] Adriaanse MC, Drewes HW, van der Heide I, Struijs JN, Baan CA (2016) The impact of comorbid chronic conditions on quality of life in type 2 diabetes patients. Qual Life Res 25(1):175–182. 10.1007/s11136-015-1061-0 26267523 PMC4706581

[25] Iglay K, Hannachi H, Howie PJ, Xu J, Li X, Engel SS, Moore LM, Rajpathak S (2016) Prevalence and co-prevalence of comorbidities among patients with type 2 diabetes mellitus. Curr Med Res Opin 32(7):1243–1252. 10.1185/03007995.2016.1168291 26986190

[26] Holloszy JO (2005) Exercise-induced increase in muscle insulin sensitivity. J Appl Physiol 99(1):338–343. 10.1152/japplphysiol.00123.2005 16036907

[27] Petersen KF, Shulman GI (2002) Pathogenesis of skeletal muscle insulin resistance in type 2 diabetes mellitus. Am J Cardiol 90(5A):11–18. 10.1016/S0002-9149(02)02554-7 12231074

[28] Dos Santos VR, Antunes M, Dos Santos L, Nascimento MA, Pina FL, Carneiro NH, Trindade MC, Venturini D, Barbosa DS, Cyrino ES (2024) Effects of different resistance training frequencies on body composition, muscular strength, muscle quality, and metabolic biomarkers in sarcopenic older women. J Strength Cond Res 38(4):e521–e528. 10.1519/JSC.0000000000004827 39178393

[29] Garneau L (2025) Are myokines linked to metabolic improvements with increased physical activity in subjects with type 2 diabetes: how acute and chronic exercise regulate myokine secretion in this population. Université d’Ottawa, Ottawa. 10.20381/ruor-30985

[30] Son JW, Lee SS, Kim SR, Yoo SJ, Cha BY, Son HY, Cho NH (2017) Low muscle mass and risk of type 2 diabetes in middle-aged and older adults: findings from the KoGES. Diabetologia 60(5):865–872. 10.1007/s00125-016-4196-9 28102434

[31] Wang Y, Lee DC, Brellenthin AG, Sui X, Church TS, Lavie CJ, Blair SN (2019) Association of muscular strength and incidence of type 2 diabetes. Mayo Clin Proc 94(4):643–651. 10.1016/j.mayocp.2018.08.037 30871784 PMC6450733

[32] Kunutsor SK, Isiozor NM, Khan H, Laukkanen JA (2021) Handgrip strength—a risk indicator for type 2 diabetes: systematic review and meta‐analysis of observational cohort studies. Diabetes Metab Res Rev 37(3):e3365. 10.1002/dmrr.3365 32543028

[33] Fritschi C, Bronas UG, Park CG, Collins EG, Quinn L (2017) Early declines in physical function among aging adults with type 2 diabetes. J Diabetes Complications 31(2):347–352. 10.1016/j.jdiacomp.2016.06.022 27450624 PMC5191982

[34] Pal GK, Adithan C, Ananthanarayanan PH, Pal P, Nanda N, Durgadevi T, Lalitha V, Syamsunder AN, Dutta TK (2013) Sympathovagal imbalance contributes to prehypertension status and cardiovascular risks attributed by insulin resistance, inflammation, dyslipidemia and oxidative stress in first degree relatives of type 2 diabetics. PLoS ONE 8(11):e78072. 10.1371/journal.pone.0078072 24265679 PMC3827034

[35] Vukomanovic V, Suzic-Lazic J, Celic V, Cuspidi C, Petrovic T, Ilic S, Skokic D, Morris DA, Tadic M (2019) Association between functional capacity and heart rate variability in patients with uncomplicated type 2 diabetes. Blood Press 28(3):184–190. 10.1080/08037051.2019.1586431 30836775

[36] Moazzeni SS, Karimi Toudeshki K, Ghorbanpouryami F, Hasheminia M, Azizi F, Pishgahi M, Hadaegh F (2023) Resting heart rate and the risk of incident type 2 diabetes mellitus among non-diabetic and prediabetic Iranian adults: Tehran lipid and glucose study. BMC Public Health 23(1):2112. 10.1186/s12889-023-17022-7 37891510 PMC10605332

[37] Grassi G, Drager LF (2024) Sympathetic overactivity, hypertension and cardiovascular disease: state of the art. Curr Med Res Opin 40:5–13. 10.1080/03007995.2024.2305248 38597067

[38] Clark DJ, Patten C, Reid KF, Carabello RJ, Phillips EM, Fielding RA (2011) Muscle performance and physical function are associated with voluntary rate of neuromuscular activation in older adults. J Gerontol A Biol Sci Med Sci 66:115–121. 10.1093/gerona/glq153 20829294 PMC3011959

