# Supplementary material for: Association between physical function and type 2 diabetes risk in older adults: a longitudinal analysis from the KoGES-Anseong cohort
Source: Eur Geriatr Med. 2026 Jul 6;17(4):2123–31. doi: 10.1007/s41999-026-01412-2 (PMC13562105; doi:10.1007/s41999-026-01412-2)
Supplement: Supplementary file 1 [file 41999_2026_1412_MOESM1_ESM.docx]

Fig 1. Flow chart of population inclusion and exclusion

**Efforts to Minimize Bias**

To minimize potential measurement bias, all objective variables were assessed by trained personnel using standardized protocols and validated equipment. Most covariates were objectively measured or derived from laboratory tests, reducing the likelihood of misclassification. Only a limited number of variables, such as physical activity, smoking, and alcohol use, were obtained via structured interviews. These interviews were conducted face-to-face and reviewed by trained interviewers to ensure consistency and completeness of responses. Therefore, the risk of information bias due to self-reporting was considered minimal.
